# Supplementary material for: Automatically assembling a full census of an academic field
Source: PLoS One. 2018 Aug 29;13(8):e0202223. doi: 10.1371/journal.pone.0202223 (PMC6114776; doi:10.1371/journal.pone.0202223)
Supplement: S1 Table — Counts of faculty directory HTML formats among PhD-granting CS departments in our sample. (PDF) [file pone.0202223.s001.pdf]

Automatically assembling a full census of an academic field

Allison C. Morgan[1*], Samuel F. Way[1], Aaron Clauset[1,2,3]

1 Department of Computer Science, University of Colorado, Boulder, CO, USA
2 BioFrontiers Institute, University of Colorado, Boulder, CO, USA
3 Santa Fe Institute, Santa Fe, NM, USA

* allison.morgan@colorado.edu

Supporting information

S1 Table. Distribution of HTML formats. Counts of faculty directory HTML formats among PhD-granting CS departments in our sample

total	divs	tables	lists	articles
205	100	80	24	1
