# Supplementary material for: Automatically assembling a full census of an academic field
Source: PLoS One. 2018 Aug 29;13(8):e0202223. doi: 10.1371/journal.pone.0202223 (PMC6114776; doi:10.1371/journal.pone.0202223)
Supplement: S2 Appendix — (PDF) [file pone.0202223.s003.pdf]

Automatically assembling a full census of an academic field

Allison C. Morgan[1*], Samuel F. Way[1], Aaron Clauset[1,2,3]

1 Department of Computer Science, University of Colorado, Boulder, CO, USA
2 BioFrontiers Institute, University of Colorado, Boulder, CO, USA
3 Santa Fe Institute, Santa Fe, NM, USA

* allison.morgan@colorado.edu

Supporting information

S2 Appendix. Features of a faculty directory. In addition to the stemmed words from directory and non-directory web pages, derived features of our random forest classifier included names, email addresses, phone numbers and titles. The complete feature set for our directory classifier can be found online at `https://github.com/allisonmorgan/academic_census`.
