# Supplementary material for: Automatically assembling a full census of an academic field
Source: PLoS One. 2018 Aug 29;13(8):e0202223. doi: 10.1371/journal.pone.0202223 (PMC6114776; doi:10.1371/journal.pone.0202223)
Supplement: S4 Appendix — (PDF) [file pone.0202223.s005.pdf]

Automatically assembling a full census of an academic field

Allison C. Morgan[1*], Samuel F. Way[1], Aaron Clauset[1,2,3]

1 Department of Computer Science, University of Colorado, Boulder, CO, USA
2 BioFrontiers Institute, University of Colorado, Boulder, CO, USA
3 Santa Fe Institute, Santa Fe, NM, USA

* allison.morgan@colorado.edu

Supporting information

S4 Appendix. Keywords in TTT and non-TTT titles. [“professor’, “faculty”, “tenure”, “people”, “full-time”, “assistant”, “associate ”, “director”, “chair”, “president”, “asst.”, “prof.”, “assoc.”, “personnel”, “professeure”, “professeur”, “research”, “visiting”, “practice”, “adjunct”, “secretary”, “admin”, “lecturer”, “emerit”, “affiliat”, “industry”, “part-time”, “instructor”, “specialist”, “advisor”, “manager”, “retire”, “courtesy”, “technical”, “post-doc”, “postdoc”, “staff”, “guest”, “collab”, “coordinat”, “develop”, “support”, “chancellor”, “scholar”, “engineer”, “joint”, “in residence”, “dir.”, “dean”, “programmer”, “analyst”, “technician”, “designer”, “co-chair”, “secondary appointment”, “teaching”, “provost”, “head”, “scientist”, “lead”, “directeur”, “phd student”, “graduate”, “fellow”]
