# Supplementary material for: Automatically assembling a full census of an academic field
Source: PLoS One. 2018 Aug 29;13(8):e0202223. doi: 10.1371/journal.pone.0202223 (PMC6114776; doi:10.1371/journal.pone.0202223)
Supplement: S5 Appendix — (PDF) [file pone.0202223.s006.pdf]

Automatically assembling a full census of an academic field

Allison C. Morgan[1*], Samuel F. Way[1], Aaron Clauset[1,2,3]

1 Department of Computer Science, University of Colorado, Boulder, CO, USA
2 BioFrontiers Institute, University of Colorado, Boulder, CO, USA
3 Santa Fe Institute, Santa Fe, NM, USA

* allison.morgan@colorado.edu

Supporting information

S5 Appendix. Keywords which should not be contained in TTT titles. [“adjoint”, “techincal”, “business advisor”, “academic advisor”, “tutoring”, “manager”, “admin”, “specialist”, “support”, “staff”, “programmer”, “guest”, “developer”, “finan’, “scientist”, “researcher”, “intern ”, “lecturer”, “analyst”, “instruct”, “post”, “technici”, “technical”, “part-time”, “part time”, “ of practice”, “ of the practice”, “research professor”, “office assistant”, “research associate”, “marketing”, “research assistant”, “teaching”, “affiliate”, “acting faculty”, “specilaist”, “outreach”, “clinical”, “partnerships”, “recruitment”, “communications”, “media relations”, “collaborator”, “visiting”, “practice”, “adj ”, “senior fellow”, “research fellow”, “avionics”, “commercialization”, “comm ”, “project director”, “adjt”, “research engineer”, “it director”, “lab director”, “managing director”, “student success”, “status-only”, “librarian”, “visitng”, “secondary faculty”, “lecturer”, “emeritus”, “hourly”, “assistant to the ”, “adjunct faculty”, “pullman bremerton everett adjunct”, “adjunct and courtesy”, “joint / courtesy faculty”, “courtesy appointment”, “secondary appointment”, “assistant adjunct professor”, “secretary”, “retired”, “emerita”, “in residence”, “program assistant”, “industry”, “emeriti”, “doctoral fellow”]
